# DNA Lesions Induced by Replication Stress Trigger Mitotic Aberration and Tetraploidy Development

**DOI:** 10.1371/journal.pone.0008821

**Published:** 2010-01-21

**Authors:** Yosuke Ichijima, Ken-ichi Yoshioka, Yoshiko Yoshioka, Keitaro Shinohe, Hiroaki Fujimori, Junya Unno, Masatoshi Takagi, Hidemasa Goto, Masaki Inagaki, Shuki Mizutani, Hirobumi Teraoka

**Affiliations:** 1 Department of Pathological Biochemistry, Medical Research Institute, Tokyo Medical and Dental University, Tokyo, Japan; 2 Biochemistry Division, National Cancer Center Research Institute, Tokyo, Japan; 3 Department of Pediatrics and Developmental Biology, Tokyo Medical and Dental University Graduate School, Tokyo, Japan; 4 Division of Biochemistry, Aichi Cancer Center Research Institute, Nagoya, Japan; Roswell Park Cancer Institute, United States of America

## Abstract

During tumorigenesis, cells acquire immortality in association with the development of genomic instability. However, it is still elusive how genomic instability spontaneously generates during the process of tumorigenesis. Here, we show that precancerous DNA lesions induced by oncogene acceleration, which induce situations identical to the initial stages of cancer development, trigger tetraploidy/aneuploidy generation in association with mitotic aberration. Although oncogene acceleration primarily induces DNA replication stress and the resulting lesions in the S phase, these lesions are carried over into the M phase and cause cytokinesis failure and genomic instability. Unlike directly induced DNA double-strand breaks, DNA replication stress-associated lesions are cryptogenic and pass through cell-cycle checkpoints due to limited and ineffective activation of checkpoint factors. Furthermore, since damaged M-phase cells still progress in mitotic steps, these cells result in chromosomal mis-segregation, cytokinesis failure and the resulting tetraploidy generation. Thus, our results reveal a process of genomic instability generation triggered by precancerous DNA replication stress.

## Introduction

Genomic instability is observed in most cancer cells [1]. In the earliest stages of cancer development, cells exhibit DNA lesions, which are characterized as precancerous DNA lesions and are induced by DNA replication stress with the accelerated cell cycle progression as the results of oncogene acceleration or of aberrant growth activation [2], [3]. During these stages, although anti-cancer barrier reactions including cell cycle arrest and inductions of senescence and apoptosis are also competitively activated to block the tumorigenesis step progression [2], [3], genomic instability is subsequently started to appear prior to the development of cancer [2], [3]. However, the process by which precancerous lesions cause genomic instability remains unclear.

The most common types of genomic instability in cancer cells are alterations in the number of chromosomes, i.e., aneuploidy [4]. Aneuploidy is suggested to develop via unstable intermediates of tetraploidy [5], [6]. In addition, tetraploidy even contributes to tumourigenesity *in vivo* [7]. Therefore, the process to generate tetraploidy must be a critical step for the development of many cancers. Furthermore, consistent with the hypothesis of aneuploidy development via unstable tetraploidy intermediates, cancer cells with chromosomal instability show the characteristics of continuous alteration in chromosomal status, highlighting the question for the initiation and the induction of tetraploidy.

Although it is elusive how tetraploidy is developed during cellular transformation, tetraploidy is often observed in cells lacking in the M-phase function [8], which also promotes tumourigenesis [9], [10]. Spontaneous tetraploidization is also observed in association with chromosome bridges during mitotic chromosome segregation and the resulting cytokinesis failure [11]. Since the appearance of precancerous lesions is followed by the development of genomic instability [2], [3], we hypothesized here that, prior to cellular transformation, precancerous DNA lesions are carried over into the M phase, causing mitotic aberrations, including chromosome-bridge formation to lead into tetraploidy generation, contributing cancer development (Supplementary Fig. S1).

For the above hypothesis, we investigated effects of DNA replication stress-associated lesions by oncogene acceleration or by hydroxyurea treatment as well as impacts of DNA lesions in the M phase, and also studied the immortalization process of primary mouse embryonic fibroblasts (MEFs). Here, we found that DNA replication stress-associated lesions can be transmitted into the M phase, unlike directly induced DNA double-strand breaks, resulting in successive chromosomal mis-segregation, cytokinesis failure and tetraploidy generation. Importantly, we observed that these happen during cellular immortalization, and found that senescing cells are temporarily accumulated with bi-nuclear tetraploidy, which is a form right after the tetraploidy generation, prior to the acquirement of the immortality.

## Results

### DNA Lesions Induced by Oncogenes Accumulate in the M Phase

**Figure 1:**
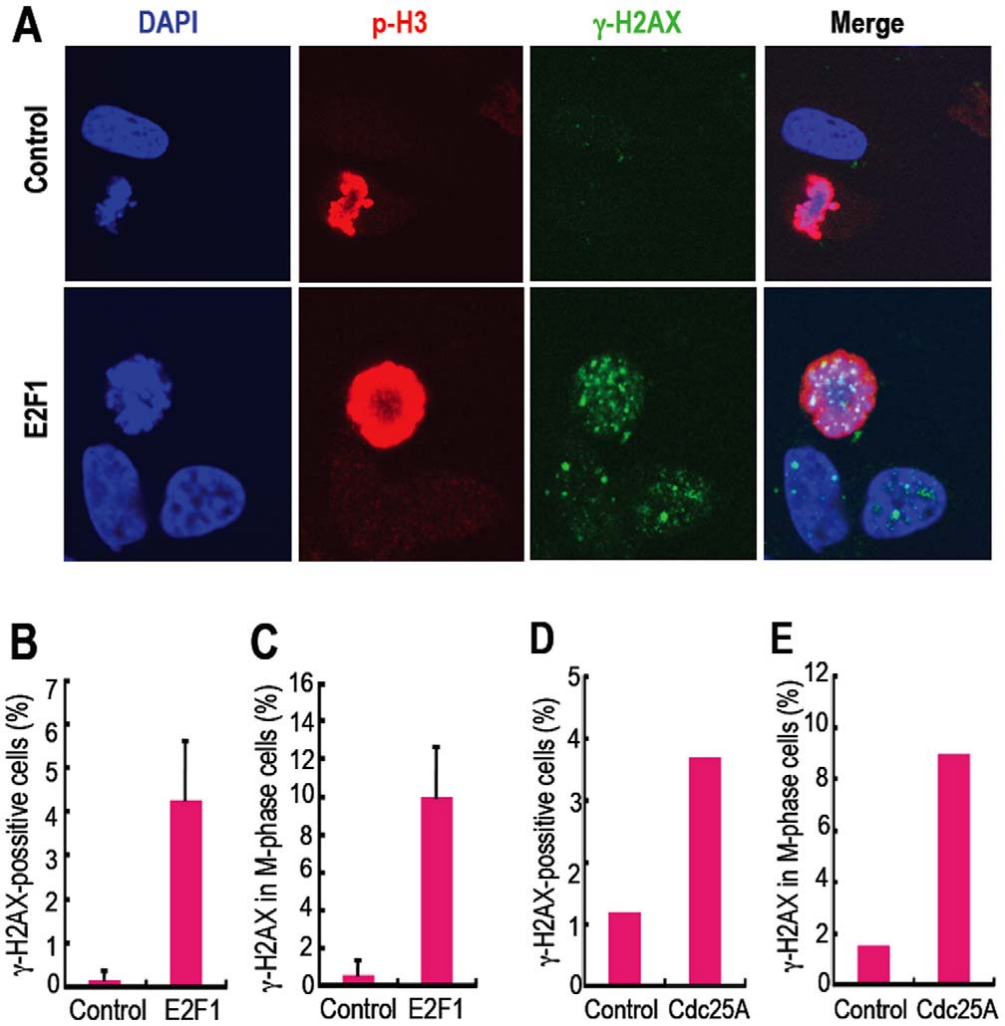
DNA lesions induced by oncogene acceleration are accumulated in the M phase. **A.** DNA lesions in the M phase were determined by a double staining of γH2AX and p-H3 after nocodazole treatment (100 ng/ml, 12 h). Using ER-*E2F1*-expressing U2OS cells, DNA lesion-carryover into the M phase was evaluated after treatment with 4-hydroxytamoxifen for 6 h (E2F1). Representative images are shown before (control) and after E2F1 activation (E2F1). **B,C.** The proportions of total γH2AX-positive cells (**B**) and γH2AX/p-H3 double-positive cells (**C**) were estimated in the cells prepared as in **A**. At least 50 cells were counted in each of 3 independent experiments. Error bars represent ± SD. **D,E.** Transient over-expression of *Cdc25A* promotes DNA lesions including the cells during mitosis. The proportions of total γH2AX-positive cells (**D**) and γH2AX/p-H3 double-positive cells (**E**) were estimated by counting at least 60 cells in **D** and 500 cells in **E**. Representative fluorescent microscope images are also shown in supplementary Fig. S2.

To test the above hypothesis (Supplementary Fig. S1), we initiated a study of DNA lesions induced by oncogenes, such as *E2F1*, because the initial stages of cancer development are mimicked by oncogene-acceleration, in which genomic instability is subsequently developed [2]. To determine the effects of the accelerated oncogene function, the spontaneous accumulation of M-phase DNA lesions was monitored with a double staining of γH2AX, a DNA-damage marker, and histone H3 phosphorylated at Ser 10 (p-H3), an M-phase marker (Fig. 1). *E2F1* acceleration caused DNA lesions in U2OS cells (Fig. 1A, B), mimicking the initial stages of cancer development as previously reported [2]. In addition, we observed that these induced DNA lesions are accumulated in mitotic cells (Fig. 1A, C). Similar results were also observed by using another oncogene *Cdc25A* in HEK293 cells (Fig. 1D, E; Supplementary Fig. S2). Thus, supporting our hypothesis (Supplementary Fig. S1), these results show that oncogenic DNA lesions are also appeared in the M phase and indicate the close correlation between mitotic precancerous DNA lesions and genomic instability development.

### Oncogene Acceleration Induces Chromosome-Bridge and Aneuploidy

**Figure 2:**
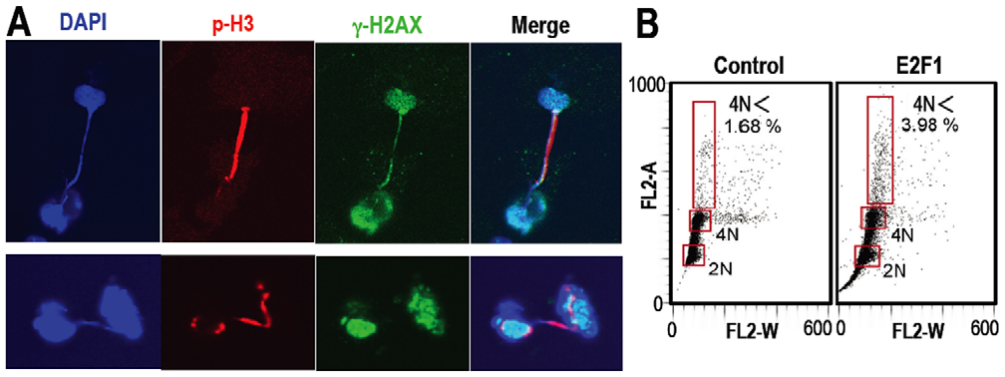
E2F1 acceleration generates chromosome bridge and aneuploidy. **A.** After E2F1 activation as in Figure 1A, chromosome bridges were often observed with the generated DNA lesions by E2F1 activation. Representative images are shown. B. Cells containing more than 4N DNA content were detected by flow cytometry in the cells treated as in Figure 1A. The proportions of cells with DNA content of 2N, 4N and more (4N<) are indicated by red squares. The percentages of 4N< cells are indicated. The sub-G1 fraction was also observed in E2F1-activated cells.

To explore the possible correlation between mitotic DNA lesions and the induction of genomic instability, we determined the appearance of chromosome bridges, because a recent study has shown that spontaneous tetraploidization is triggered by chromosome bridges [11], though it remains elusive how chromosome bridges are induced. After *E2F1* acceleration, we observed chromosome bridges (Fig. 2A) concomitantly with the elevation of polyploidy fraction (Fig. 2B). Intriguingly, such a chromosome bridge was observed with γH2AX signal on the chromosome (Fig. 2A), indicating the involvement of DNA lesions in the chromosome bridge formation. Taken together, these results support our hypothesis (Supplementary Fig. S1) and indicate that precancerous DNA lesions induced by oncogenes trigger chromosome bridges during mitosis and induce genomic instability. However, oncogene activation primarily accelerates S-phase entry, thereby the resulting DNA lesions are primarily associated with DNA replication stress in the S phase [2]. Here, an important question arose, if the observed M-phase lesions possibly transmit into the M phase from the S phase with the bypass of cell cycle checkpoints.

### DNA Replication Stress-Associated Lesions Transmit into the M Phase

**Figure 3:**
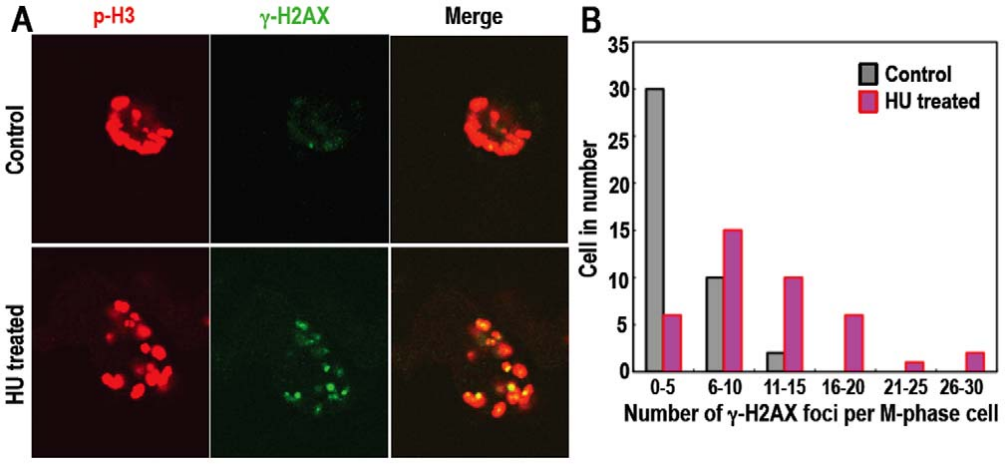
DNA lesions induced by replication stress are transmitted into the M phase. **A,B.** Using the normal human fibroblast SuSa, the carryover of DNA replication stress-associated lesions was determined after the transient treatment of 1 mM HU for 24 h and the subsequent nocodazole block as in Methods. The representative images (**A**) and the number of γH2AX foci per cell (**B**) are shown. The number of γH2AX foci was counted from 42 control cells and 40 replication stress-induced cells.

To directly determine the potential of DNA lesion-carryover generated by DNA replication stress in the S phase, we transiently treated the normal human fibroblast SuSa with hydroxyurea (HU) to cause replication fork stalling and the resulting DNA double-strand breaks. After the transient replication stress, γH2AX foci were evidently increased in the subsequent M phase (Fig. 3A,B), showing that DNA lesions induced by replication stress actually transmit into the M phase. However, an important question remains: How can DNA lesions generated by replication stress be carried over into the M phase, despite the existence of the firmly established intra-S and G2/M checkpoints?

**Figure 4:**
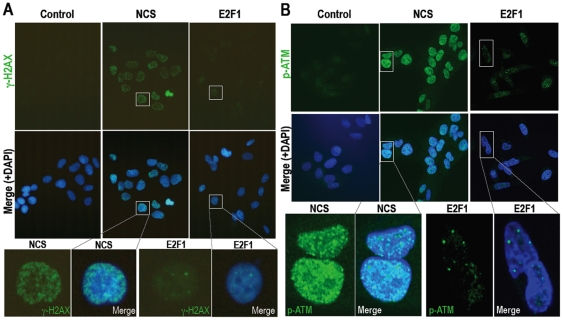
DNA replication stress causes only a small number of DNA lesions with limited ATM activation. **A,B.** Comparing cells activated with E2F1 as in Fig. 1A and cells damaged with 100 ng/ml NCS that causes G2-phase arrest, the statuses of DNA lesions and the resulting damage checkpoint activation were determined with γH2AX foci (**A**) and phosphorylated ATM (P-ATM) foci (**B**), respectively.

Recently, DNA lesion-carryover into the M phase has been shown with fewer than 20 foci of γH2AX per nucleus in the ATM-mutated background after X-ray or γ-ray irradiation [12], implying that cell cycle checkpoints are bypassed under a small number of lesions with compromised damage checkpoint response. To determine the status of DNA lesions and checkpoint activation, we compared γH2AX signals and phosphorylated ATM (p-ATM) signals after *E2F1* acceleration with those of the radiomimetic agent neocarzinostatin (NCS) that causes G2-arrest. While NCS causes γH2AX and the resulting p-ATM foci in the entire nucleus, *E2F1* acceleration was found to cause only very limited γH2AX (Fig. 4A) and the resulting much weaker and limited p-ATM foci (Fig. 4B), indicating only local checkpoint activation. Taken together, our results suggest that DNA lesions induced by replication stress under *E2F1* acceleration, unlike directly induced DNA double-strand breaks, impact a small number of DNA lesions, resulting in limited damage checkpoint response, bypass of cell-cycle checkpoints and DNA lesion-carryover into the M phase.

### DNA Lesions in the M Phase Cause Cytokinesis Failure

**Figure 5:**
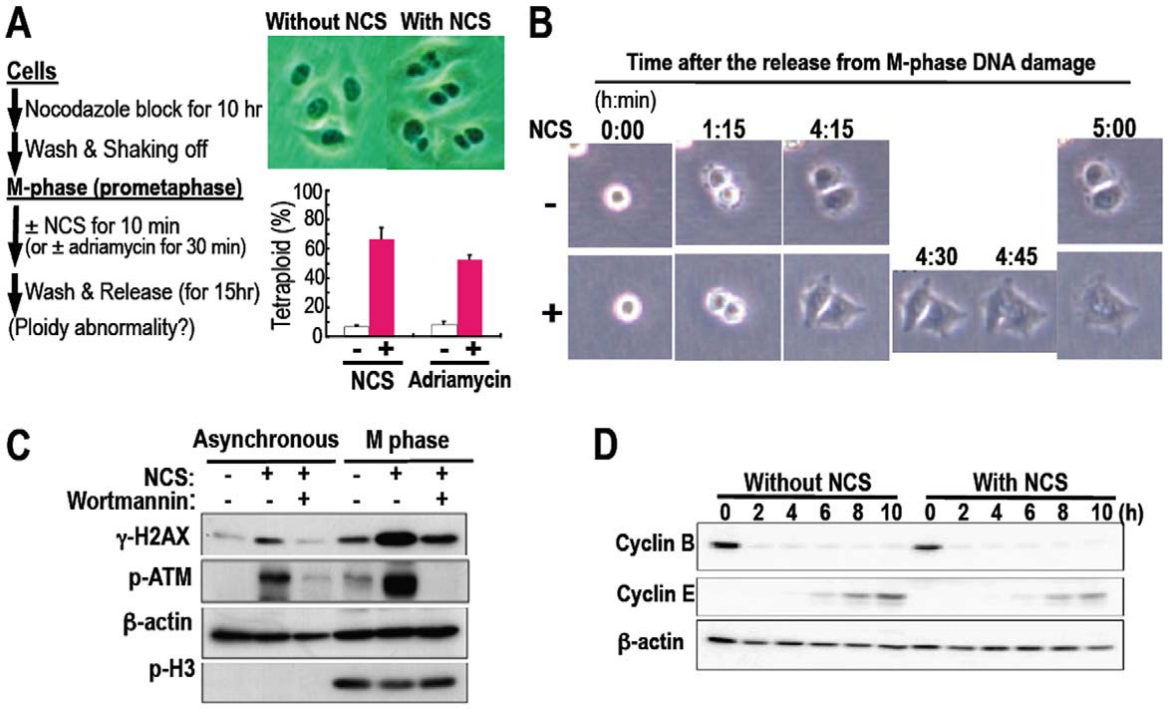
Damaged mitotic cells still proceed into cytokinesis with failure, resulting in tetraploidy generation. **A.** The tetraploidy generation was determined as in the scheme with DNA damage in the M phase. The fraction of tetraploidy was quantified from at least 100 cells in each of 3 independent experiments. Error bars in the graphs represent ± SD. **B.** Time-lapse imaging analyses were performed for the damaged cells as in **A** after the release. The representative images are displayed at the indicated time points. These results are also shown with movies [Supplementary movies S1, S3 (control) and S2, S4 (damaged with NCS)]. **C.** Mitotic cells still show the functional activation of DNA damage checkpoint factors, although cells still proceed into the G1 phase as in **D**. When indicated, the cells were incubated with 40 µM wortmannin for 1 h before NCS treatment. **D.** M-phase exit and G1-phase entry of the damaged cells as in **A** were determined with cyclins B and E as M- and G1-phase markers, respectively, after the release.

Another question remains: How do mitotic cells readily respond to DNA lesions that are carried over into the M phase? Despite the numerous studies on DNA damage response, only a few studies of those have been reported for the mitotic cells, showing M-phase specific DNA damage checkpoints [13], [14]. Interestingly, one of these has reported tetraploidization with ionizing radiation in prometaphase HeLa cells [13]. Here, we found that such tetraploidization is a common phenomenon, independent of damaging sources (Fig. 5A) and cell types, including U2OS, WI-38, and MEFs (Supplementary Fig. S3), as long as cells existed in the M phase (Supplementary Fig. S4). These showed completely different responses to DNA damage in the M phase.

For the detailed study on tetraploidization with DNA lesions in mitotic cells, we used time-lapse imaging (Fig. 5B; Supplementary Movies S1, S2, S3, S4) and found that the damaged cells failed to complete cytokinesis and subsequently developed tetraploidy (Fig. 5B lower panels between 4:30 and 5:00). Importantly, such a cytokinesis failure was observed in the majority of cells (Supplementary Movie S4; Fig. 5A), and those still replicated DNA (Supplementary Fig. S5). Furthermore, despite the activation of DNA damage checkpoint proteins, including H2AX, ATM and Chk2 (Fig. 5C; Supplementary Fig. S6), damaged M-phase cells still exited from the mitotic phase and entered into the G1 phase, based on monitoring cyclins B and E as M- and G1-phase markers, respectively (Fig. 5D), indicating the dysfunctional DNA damage checkpoint during mitosis. Since cells had already exited from the metaphase, the spindle assembly checkpoint could not be responsible for DNA damage. In fact, a spindle assembly checkpoint factor, BubR1, exhibited normally (Supplementary Fig. S7). Thus, DNA damage checkpoints are not fully functional during mitosis, even if they exist [13], [14].

**Figure 6:**
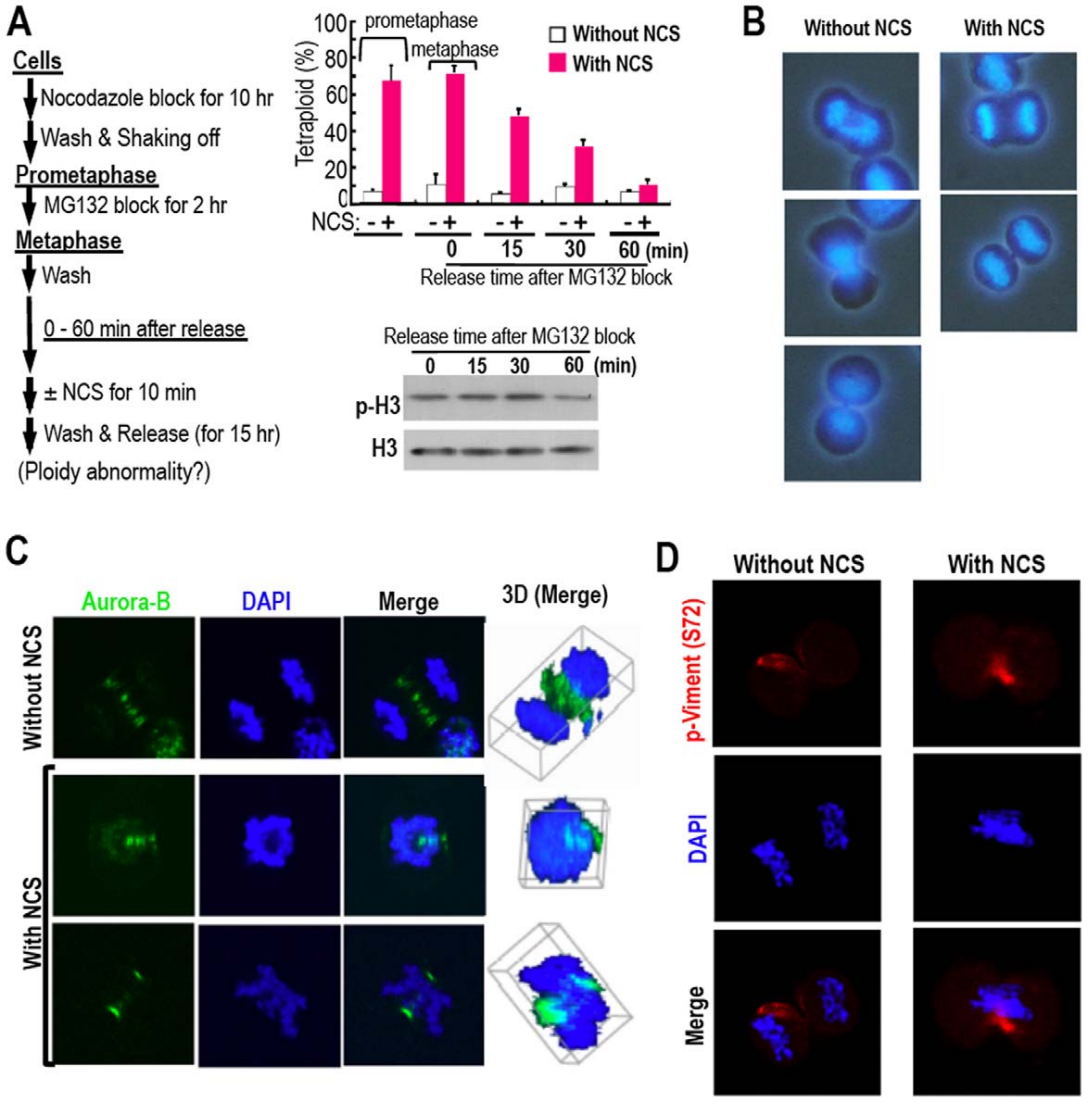
Mitotic DNA lesions cause chromosomal mis-segregation and a disruptive spindle mid-zone, followed by tetraploidy generation. **A**. DNA damage causes tetraploidy generation during metaphase but not after the onset of anaphase. The binuclear tetraploidy generation was assessed for the cells in metaphase or later as in the scheme using prometaphase cells prepared as in Figure 5A. Data were determined as in Figure 5A with at least 60 cells in each. **B.** To determine the chromosomal mis-segregation, cells damaged as in Figure 5A were released for 1 h and stained with DAPI. Images of the cells are at different mitotic stages. **C.** To determine the status of spindle midzone structure, cells damaged as in **A** were released for 1.5 h and analyzed for Aurora-B localization. Three-dimensional images are also displayed. **D.** Vimentin, a target of Aurola B, is activated with the phosphorylation status of vimentin at Ser 72 even under the chromosome mis-segregation, in which cells were treated with NCS for 1.5 h.

In addition, tetraploidization was also observed in metaphase cells but was significantly lowered 15 min after metaphase release (Fig. 6A), suggesting the involvement of chromosome segregation, because chromosome segregation starts at the onset of the anaphase. In fact, the cells damaged in the prometaphase showed incomplete chromosome segregation (Fig. 6B). Furthermore, such chromosomal mis-segregation disrupted the spindle midzone structure, including Aurora-B localization (Fig. 6C), which is the essential conformation for cytokinesis [15], [16]. Here, Aurora-B kinase was still active (Fig. 6D), though. A recent study has shown that Aurora-B functions to protect tetraploidization as an abscission checkpoint, although this is not the perfect block, either [11]. Taken together, these findings indicate that DNA lesions in the M phase cause a chromosome bridge and disrupt the spindle midzone structure, risking cytokinesis failure and tetraploidization.

### MEFs Are Immortalized with Tetraploidy

**Figure 7:**
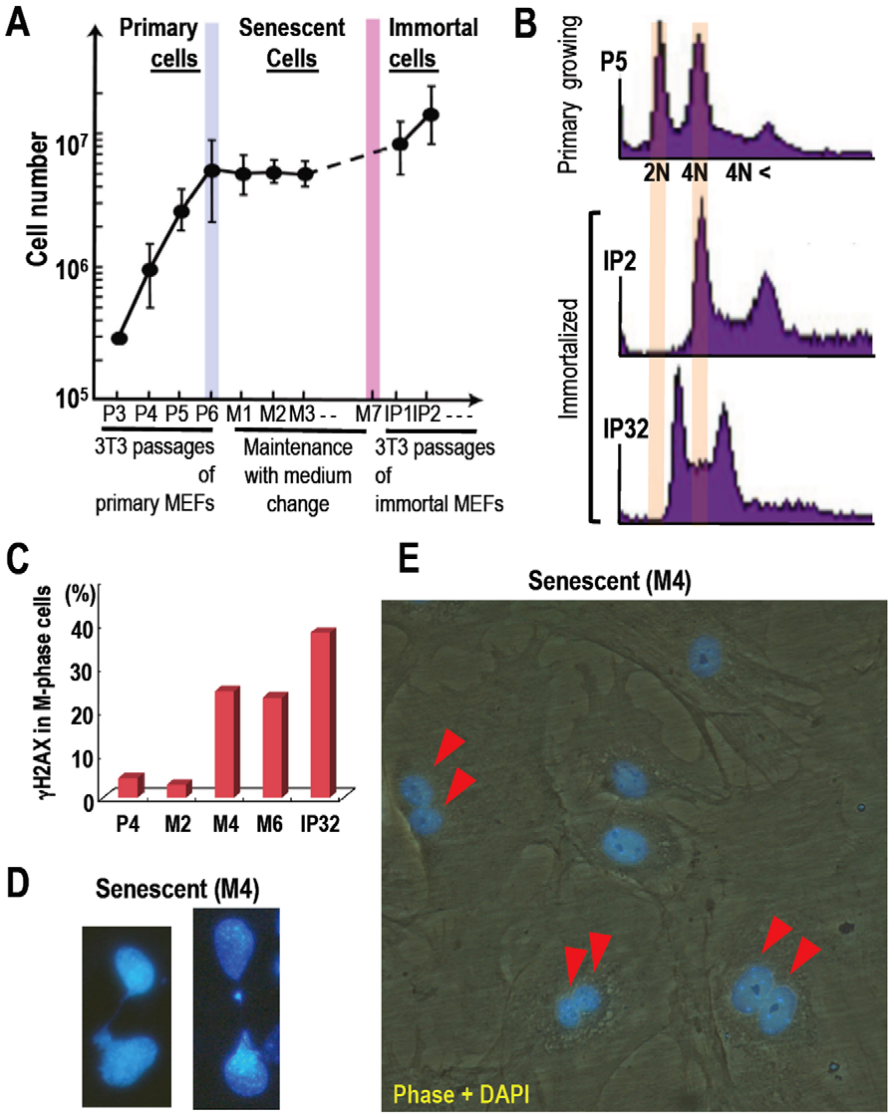
Senescing MEFs develop tetraploidy in association with mitotic DNA lesions and are accumulated with tetraploidy status before acquiring immortality. **A.** Growth curve of MEFs showed following 3 phases: primary growth (P3-P6); senescence (M1-M7); immortal growth (IP1-) phases. MEFs were passed under the 3T3 protocol or maintained with medium change once in 3 days. **B.** To determine the chromosomal status, either diploid or tetraploid/aneuploid, the chromosome contents were analyzed for primary growing (P5) and early (IP2) and late (IP32) immortalized MEFs. **C.** To determine the status of mitotic DNA lesions during the MEFs life cycle, MEFs in each step were determined by a double staining of γH2AX and phoshorylated H3 (p-H3) after nocodazole treatment (100 ng/ml, 12 h). γH2AX/p-H3 double positive fractions were determined at the indicated stages. **D.** Chromosome bridges were observed at M4. Images are representative. **E.** The image is representative at M4, showing the accumulation of cells with bi-nuclear tetraploidy. Arrowheads indicate cells with bi-nuclear tetraploid (Red arrowheads).

As described above, DNA lesions induced by oncogenes, which could act as precancerous DNA lesions, are possibly carried over into the M phase, causing a chromosome-bridge and the resulting cytokinesis failure with tetraploidy generation. To confirm whether such scenario is really the case during spontaneous cell immortalization, we tested during the process of MEF-immortalization, (1) because MEFs are immortalized with the mutation in the Arf/p53 module similar to cancer development [17], (2) because primary MEFs often develop tetraploidy prior to immortalization, and (3) because senescing cells are known to spontaneously accumulate unrepairable DNA lesions [18], as potentially precancerous DNA lesions. We cultured growing-MEFs under the 3T3 protocol [19] and maintained senescing-MEFs with medium change (Fig. 7A). As well established, MEFs initially showed primary growth and then slowed down during senescene, which was followed by development of immortality. Intriguingly, all immortalized MEFs at early steps (IP2) were completely tetraploidy (Fig. 7B), implying that tetraploidization is the key step for MEF-immortalization. In addition, these immortalized MEFs lost the function of p53 accumulation in response to DNA damage, whereas senescing MEFs as well as primary growing MEFs showed p53 accumulation after DNA damage (Supplementary Fig. S8). This suggests that the induction of mutations is also associated with genomic instability development during immortality acquirement, although it is still unclear how the mutations are induced.

### Spontaneous MEF-Tetraploidization Is Associated with M-Phase DNA Lesions and Chromosome-Bridge

To determine the possible association between M-phase DNA lesions and tetraploidy generation during MEF-imortalization, we examined the status of DNA lesions in the M-phase cells in each stage during the lifecycle of MEFs (Fig. 7C). Importantly, DNA-lesions in the mitotic cells were observed in the rarely growing senescent (M4 and M6) as well as in immortalized MEFs (IP32), but not in MEFs under primary growth (P4) or early senescence (M2) (Fig. 7C). These results indicate that spontaneous DNA lesions in the M phase starts to appear in the rarely growing senescent MEFs prior to the acquirement of immortality. Importantly, M-phase DNA lesions at M4 concurrently appeared with chromosome-bridge (Fig. 7D) and bi-nuclear tetraploidy (Fig. 7E). These results support that DNA lesions trigger the chromosome-bridge and the resulting tetraploidy generation during MEF-immortalization, because the observed bi-nuclear tetraploidy is a primary and transient status right after the development until the following M phase, in which daughter chromosomes assemble in a common metaphase plate to lead into tetraploidy with a single nucleus in the subsequent G1 phase [11]. Importantly, these results indicate that tetraploidy-generation associated with mitotic DNA-lesions is also the case during MEF immortalization. Furthermore, the resulting immortal MEFs (IP2) were totally tetraploidy (Fig. 7B), indicating that the tetraploidization step is critical for acquiring immortality. In addition, DNA lesions spontaneously accumulating in senescing cells act qualitatively similar to the lesions induced by oncogenes.

After immortalization, MEFs were mostly γH2AX-positive and continuously showed DNA lesions during mitosis (Fig. 7C), suggesting continuous genomic alterations. In fact, the continuous culture of immortalized MEFs resulted in chromosomal loss, i.e., aneuploidy, at IP32 (Fig. 7B), which is an identical characteristic to cancer cells showing continuous chromosomal instability [1]. These results also support the previously proposed hypothesis, i.e., aneuploidy generation via the unstable tetraploidy [5], [6]. However, these M-phase lesions in the immortalized MEFs did not trigger further polyploidy generation (Fig. 7B). Similarly, tetraploidy causes growth retardation and thereby never becomes major, although spontaneous development of tetraploidy is often observed during HeLa cell cultivation via chromosome bridges [11]. While tetraploidization with M phase-DNA lesion must be a key step for acquiring immortality, the impact of tetraploidization is likely to different once cells are immortalized. Nevertheless, our results suggest that, during senescing MEF immortalization, M phase-DNA lesions trigger spontaneous development of tetraploidy.

### Tetraploidy Development in MEFs Is Accelerated by DNA Replication Stress

**Figure 8:**
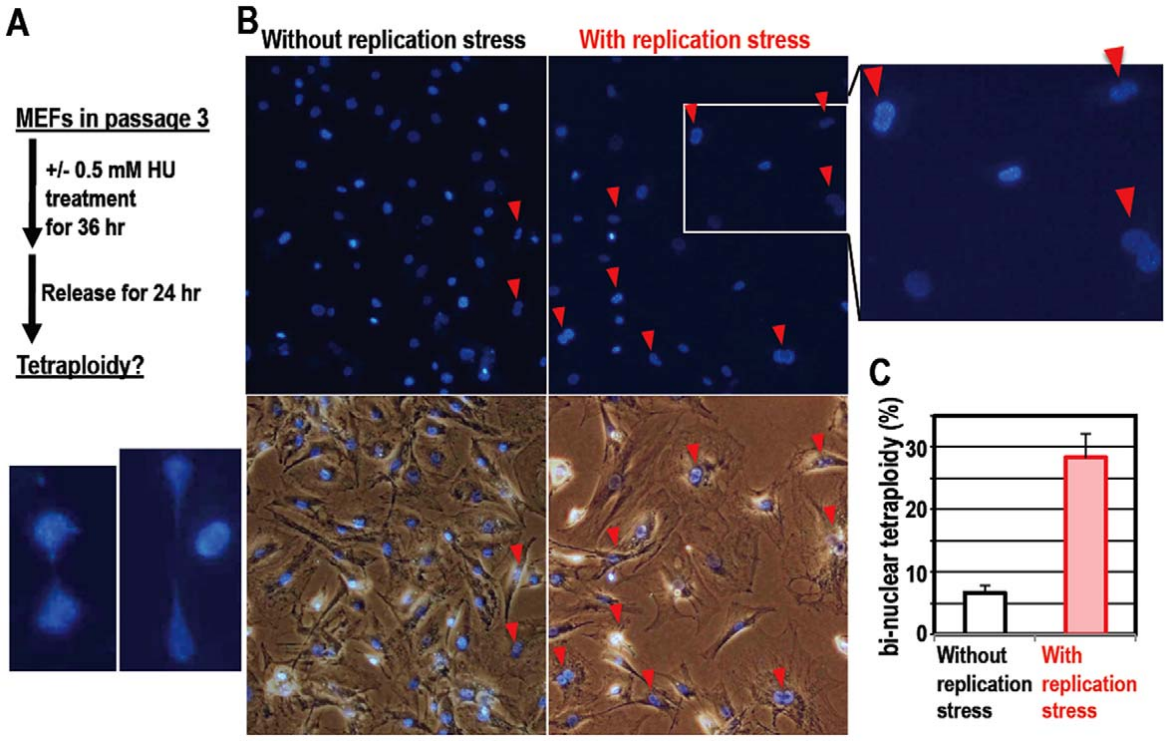
DNA replication stress induces chromosomal bridge formation and tetraploidy in early passage-primary MEFs. **A.** The effect of DNA replication stress was determined as in the scheme. After transient DNA replication stress, chromosome bridges were often observed. Representative images are shown. **B.** The images are representative with or without DNA replication stress. Arrowheads indicate cells with bi-nuclear tetraploid (red arrowheads). **C.** The proportions of total bi-nuclear cells were estimated. At least 100 cells were counted in each of 3 independent experiments.

Through above study, we showed that DNA replication stress-associated lesions are transmitted into the M phase, that DNA lesions during mitosis cause tetraploidy generation, and that the identical processes are observed during the immortalization of MEFs. To directly confirm our original hypothesis (Supplementary Fig. S1), we further investigated whether tetraploidy generation could be directly induced by DNA replication stress in the pre-immortalizing MEFs (P3). Consistent with our above results, transient replication stress induced chromosome-bridge formation (Fig. 8A) and bi-nuclear tetraploidy accumulation (Fig. 8B,C) even in early passage MEFs (passage 3). Furthermore, these bi-nuclear tetraploidy MEFs were also subsequently immortalized. These indicate that DNA lesions induced by replication stress mediate tetraploidy generation in association with chromosome bridge formation during the acquirement of immortality.

## Discussion

Cancer is a disease associated with genomic instability, which develops prior to tumor formation. Cells in the initial stages of cancer development exhibit precancerous DNA lesions and the competitive barrier responses [2], [3]. Such stages are followed by the development of genomic instability [2], [3], although it was elusive how and why genomic instability could develop in such situation. Our results showed one of the processes in developing genomic instability by precancerous DNA lesions, in which the lesions are carried over into the M phase and cause chromosomal mis-segregation and cytokinesis failure, resulting in tetraploidy generation. Such a conclusion is based on the following mechanistic findings: DNA replication stress-associated lesions, which are induced by oncogene acceleration, can be carried over into the M phase; DNA lesions in mitotic cells cause chromosomal mis-segregation and the resulting cytokinesis failure.

Genomic instability is categorized in chromosomal instability (CIN) and microsatellite instability (MIN) [4]. While MIN is mostly characterized by mismatch repair (MMR) deficiency, CIN is usually MMR proficient. Our study revealed a process of CIN generation, especially tetraploidy/aneuploidy. Similarly, a previous study has shown that chromosomal translocation is also observed with G2-phase DNA lesions in the following G1 phase [20]. Thus, aberrant chromosomal segregation induced by DNA lesions might generally cause chromosomal alteration with the resulting loss of genomic homeostasis, which is also consistent with the observation of chromosomal loss in association with M-phase DNA lesions during the continuous culture of the immortalized MEFs (Fig. 7B).

Consistent with ageing-associated cancer-risk elevation, our results suggest that spontaneous DNA lesions accumulated in senescent cells during MEF immortalization act as precancerous DNA lesions, similar to the lesions induced by oncogene acceleration. Our results also show that DNA lesions generated by DNA replications stress are cryptogenic due to the limited impact on DNA lesions and the checkpoint activation, and that these lesions therefore induce genomic instability after the transmission into the M phase. Such a conclusion, i.e., genomic instability induction by DNA replication stress, is supported by the evidence of cancer predisposition with defective homologous recombination in BRCA1, BRCA2 and BLM helicase mutants [21]–[23], because DNA replication stress-associated lesions are primarily the target of homologous recombination.

Here we observed that the escape of G2/M checkpoint with DNA lesions triggers tetraploidy development. Contrary, previous reports showed that the identical escape of G2/M checkpoint results in the mitotic catastrophe cell death [24]–[27]. How the identical DNA lesions could induce completely different effects? Although the mechanistic discrimination is unclear, so far the differences underlie if the cells are in immortal or pre-immortal. In cancer cells, cells with aneuploidy were accumulated after G2/M checkpoint-escape and after the appearance of chromosome bridge (Fig. 1,2). But such aneuploidy accumulates transiently and never come up to major, which was also shown in a previous HeLa cell study [11]. Such transient accumulation of aneuploidy coincided with the increase in sub-G1 fraction (Fig. 2B), suggesting the eventual death induction. In contrast, pre-immortal senescing cells are accumulated with bi-nuclear tetraploidy in association with the escape of G2/M checkpoint, and eventually acquire the immortality, which are totally tetraploidy. In fact, mitotic catastrophe-associated death induction has been mainly studied with the immortalized cells mostly in cancer cell lines, which are described as a goal of cancer therapies. Contrary, somehow pre-immortal cells are resistant to the identical DNA lesions and survive, contributing the development of the immortality.

Prior to acquiring immortality, senescing MEFs are accumulated with a bi-nuclear phenotype that is a primary and transient form of tetraploidy, indicating that such tetraploidy generation in senescing cells is the major event in these stages in association with M-phase DNA lesions, aberrancy in chromosomal segregation and cytokinesis failure. Since immortalized MEFs are totally tetraploidy, these steps must be critical for immortalization. It has been shown that immortalized MEFs are mutated in the Arf/p53 module [17]. We also observed that the Arf/p53 module responds normally in senescing MEFs unlike that in immortalized MEFs (Supplementary Fig. S8), suggesting that the selective pressure of mutants is also coupled with acquiring immortality and tetraploidy development. Here we showed the mechanistic steps of MEFs immortalization, which share with the process of cancer development in many aspects. However, unlike MEFs, primary human cells usually do not show such spontaneous transformation. Difference in MEFs and human cells is mainly because MEFs express TERT and suffer from accelerated growth stimulation with 10% fetal bovine serum, whereas primary human cells require hTERT and the additional acceleration of oncogenes such as Myc, Ras etc. for the immortalization [28], [29]. Importantly, our results suggest that the trigger for immortality acquirement-associated development of genomic instability is the precancerous DNA replication stress with oncogene acceleration or with senescence-associated repair deficiency with continuous growth stimulation.

## Materials and Methods

### Cell Culture, Oncogene Induction, Cell Synchronization, Cell Damage and Replication Stress Induction

Cancer cell lines and normal human fibroblast SuSa were cultured as previously described [30]. MEF cells were prepared as previously described [19]. MEFs were cultured under 3T3 passage protocol [19], in which 3×10^5^ MEFs were passed in 6-cm dishes every 3 days using 10% fetal bovine serum containing DMEM (during P1-P6 and after IP1), otherwise maintained with medium-change under the same medium conditions every 3 days (during M1-M7). ER-E2F1 expressing U2OS cells were treated with 4-hydroxytamoxifen (300 nM) as previously described [31]. For transient expression of *Cdc25A*, *Cdc25A* cDNA was inserted into pIREShyg2 vector (Clontech Laboratories, Palo Alto, CA). The Cdc25A expression vector, empty vector, or none was then transfected into HEK293 cells with FuGENE6. Prometaphase cells were prepared as previously reported [32]. For the preparation of metaphase cells, prometaphase cells were further incubated with 10 µM MG132 for 2 h [33]. These synchronization and chromosome contents were determined with flow cytometry as previously described [32]. DNA double-strand breaks were directly induced by 100 ng/ml NCS (Pola Pharma, Tokyo, Japan) for 10 min or by 2.5 µM adriamycin for 1 h. Induced DNA lesions were detected by γH2AX, which were confirmed with comet assay after NCS treatment (Supplementary Fig. S9). For DNA replication stress-associated DNA-LCM study, SuSa cells were transiently treated with 1 mM HU for 24 h and then released in 10 % FBS DMEM with 20 ng/ml nocodazole for 10 h.

### Antibodies, Immunostaining and Western Blotting

Antibodies against γH2AX (JBW301, Upstate Biotechnology) and phospho-histone H3 (Ser 10) (Upstate Biotechnology) were used for immnostaining and Western blot analysis. Antibodies against phospho-ATM (Ser 1981) (10H11.E12, Cell Signaling Technology), phospho-Chk2 (Thr 68) (Cell Signaling Technology), β-actin (AC-74, Sigma), histone H3 (ab1791, Abcam), cyclin B1 (GNS1, Santa Cruz Biotechnology Inc.), p53 (Pab240, Santa Cruz Biotechnology Inc.) and cyclin E (Ab-1, Calbiochem) were used for Western blot analysis. Antibodies against AIM-1 (Aurora-B) (BD Transduction Laboratories), phospho-vimentin (Ser 72) [34], BubR1 (8G1, Upstate Biotechnology) and phospho-ATM (Ser 1981) (clone 7C10D8, Rockland) were used for immunostaining. Before immunostaining with primary and secondary antibodies, cells were fixed with 4% paraformaldehyde for 10 min and permeabilized with 0.1% Triton X-100/PBS for 10 min. For confocal microscope imaging, cells were cultured on coverslips and stained as above. Other immunofluorescence images were captured with ECLIPSE TE300 inverted microscope (Nikon) or LSM510 confocal microscope (Carl Zeiss). Three-dimensional images were constructed with 1 µm-slice pictures of the cells using LSM Image Browser software. Western blot analysis was performed as previously described [32].

### Immunofluorescence and Time-Lapse Imaging

Fifteen hours after the release from M phase-DNA damage, cells were fixed with 10% neutral buffered formalin for 10 min, permeabilized with 0.3% Triton X-100/PBS for 10 min, and stained with DAPI for 5 min. Phase contrast images merged with immunofluorescence images were captured with ECLIPSE TE300 inverted microscope. Time-lapse images were acquired with Multicell-imaging incubator (Sanyo).

### Comet Assay

A comet assay was performed as previously described [32].

### Chromosome Spreads

Mitotic cells were prepared in a 6-h treatment with 20 ng/ml nocodazole and shaking-off. The collected cells were hypotonically swollen with 75 mM KCl for 15 min, and then fixed with −20°C Carnoy's solution (75% methanol/25% acetic acid) for 20 min. The fixative was changed once and the cells in Carnoy's solution were dropped onto glass slides and air-dried. The slides were stained with 4% Giemsa (Merck) solution for 10 min, washed briefly in tap water, and air-dried.

## Supporting Information

Movie S1 Movies S1-S4. For the precise investigation of the process of tetraploidy development in the M-phase cells with DNA lesions, time-lapse imaging was performed. After cells were damaged with NCS as in Fig. 5A, the damaged cells (Movies S2 and S4) or non-damaged control (Movies S1 and S3) were monitored with close-up views (Movies S1 and S2) or wide-range views (Movies S3 and S4). The images shown in Fig. 5B are from those in Movies S1 and S2.
(0.27 MB MOV)

Movie S2 Movies S1-S4. For the precise investigation of the process of tetraploidy development in the M-phase cells with DNA lesions, time-lapse imaging was performed. After cells were damaged with NCS as in Fig. 5A, the damaged cells (Movies S2 and S4) or non-damaged control (Movies S1 and S3) were monitored with close-up views (Movies S1 and S2) or wide-range views (Movies S3 and S4). The images shown in Fig. 5B are from those in Movies S1 and S2.
(0.27 MB MOV)

Movie S3 Movies S1-S4. For the precise investigation of the process of tetraploidy development in the M-phase cells with DNA lesions, time-lapse imaging was performed. After cells were damaged with NCS as in Fig. 5A, the damaged cells (Movies S2 and S4) or non-damaged control (Movies S1 and S3) were monitored with close-up views (Movies S1 and S2) or wide-range views (Movies S3 and S4). The images shown in Fig. 5B are from those in Movies S1 and S2.
(1.41 MB MOV)

Movie S4 Movies S1-S4. For the precise investigation of the process of tetraploidy development in the M-phase cells with DNA lesions, time-lapse imaging was performed. After cells were damaged with NCS as in Fig. 5A, the damaged cells (Movies S2 and S4) or non-damaged control (Movies S1 and S3) were monitored with close-up views (Movies S1 and S2) or wide-range views (Movies S3 and S4). The images shown in Fig. 5B are from those in Movies S1 and S2.
(1.09 MB MOV)

Figure S1 Hypothesis. Cells damaged with precancerous DNA lesions develop tetraploidy hypothetically via chromosomal bridges during chromosomal segregation (bottom), unlike cell division in cells without DNA lesions (top). If this is the case, generated cells with tetraploidy are primarily and transiently bi-nuclear until the following M phase, in which daughter chromosomes assemble in a common metaphase plate to lead into tetraploidy with a single nucleus in the subsequent G1 phase.
(3.03 MB TIF)

Figure S2 Transient over-expression of Cdc25A promotes DNA lesions including the cells during mitosis. Empty (control) or Cdc25A expression (Cdc25A) vectors were transfected into HEK293 cells. After cultivation for two days, cells were determined with the indicated antibodies.
(3.02 MB TIF)

Figure S3 Tetraploidy generation with DNA damage during mitosis in U2OS, WI-38 and primary MEFs. A. Cells prepared as in the experimental scheme on Fig. 5A were stained with DAPI. The arrowheads indicate bi-nuclear tetraploid cells. B. Quantification of the tetraploid cells was performed with at least 100 cells for each.
(2.99 MB TIF)

Figure S4 Cells damaged during mitosis lead to tetraploidy generation but not during interphase. HeLa cells in the M phase or without synchronization were treated as in the scheme. Unlike asynchronous cells, M phase-cells specifically develop tetraploidy after damage. Quantification of the tetraploid cells was performed with at least 100 cells for each.
(2.21 MB TIF)

Figure S5 The cells damaged in the M phase further replicate DNAs in the following S phase. A,B. After cells were damaged with NCS (A) or adriamycin (B) as in Fig. 5A, the chromosome contents of the cells after the release were analyzed by flow cytometry.
(1.10 MB TIF)

Figure S6 DNA damage checkpoint activation is durable in the M phase, but dysfunctional to induce arrest during mitosis. The activation of DNA damage checkpoint protein Chk2 in the HeLa asynchronous and M-phase cells characterized by phosphorylated histone H3 (P-H3) was analyzed for the phosphorylated form.
(0.37 MB TIF)

Figure S7 Prometaphase-DNA damage does not affect the behavior of BubR1 and the progression into the anaphase and the telophase. At 75 min after the release from NCS treatment as in the experimental scheme on Fig. 5A, the cells were stained with anti-BubR1 antibody and DAPI. For the NCS-treated cells, the mitotic stages in the anaphase and the telophase are estimated based on the degree of cell elongation.
(4.78 MB TIF)

Figure S8 Arf/p53 module mutation in the immortalized MEFs. To determine the loss of Arf/p53 module, p53 accumulation was monitored 12 h after 100 ng/ml NCS treatment at each stage of MEFs: primary growth (P4); senescence (M2); immortalized (IP2).
(0.63 MB TIF)

Figure S9 DNA lesions indicated by γH2AX were also confirmed with comet assay. DNA lesions, indicated by γH2AX in this study, were also confirmed by comet assay with the tails after NCS treatment for 15 min. Arrow heads indicate the spots with comet tails, indicating DNA damages.
(2.88 MB TIF)
