# Supplementary figures and images for: DNA Lesions Induced by Replication Stress Trigger Mitotic Aberration and Tetraploidy Development
Source: PLoS One. 2010 Jan 21;5(1):e8821. doi: 10.1371/journal.pone.0008821 (PMC2809090; doi:10.1371/journal.pone.0008821)

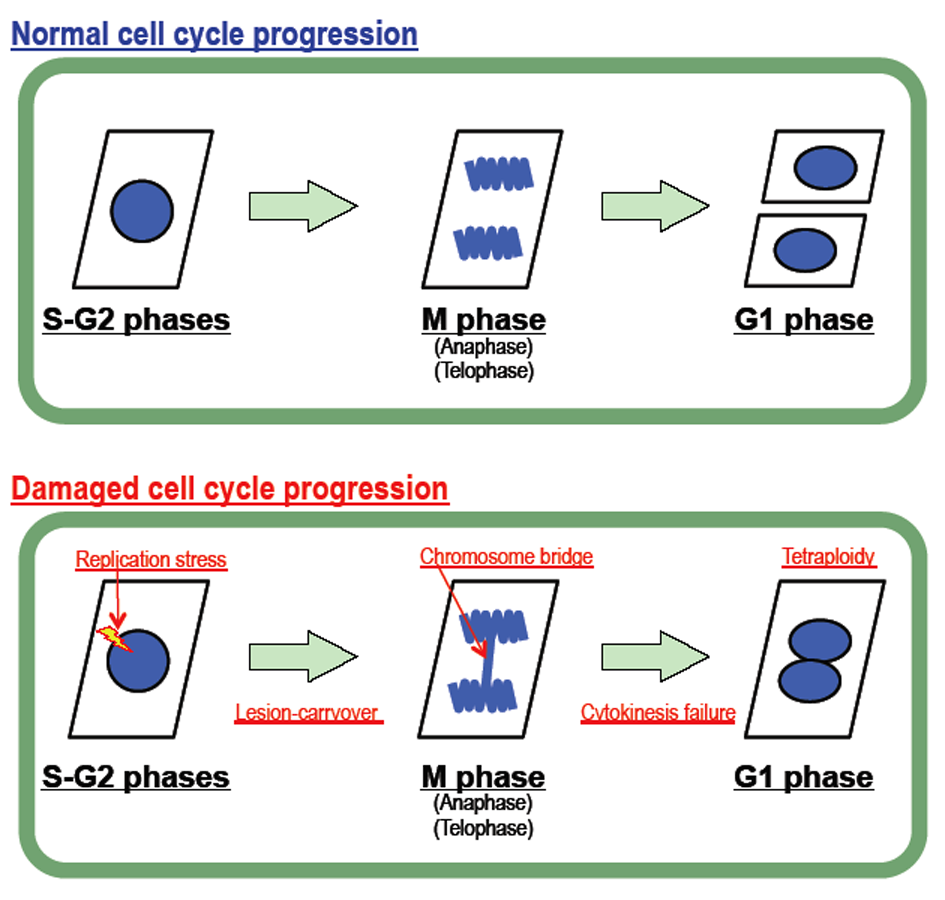

Supplement: Figure S1 — Hypothesis. Cells damaged with precancerous DNA lesions develop tetraploidy hypothetically via chromosomal bridges during chromosomal segregation (bottom), unlike cell division in cells without DNA lesions (top). If this is the case, generated cells with tetraploidy are primarily and transiently bi-nuclear until the following M phase, in which daughter chromosomes assemble in a common metaphase plate to lead into tetraploidy with a single nucleus in the subsequent G1 phase. (3.03 MB TIF) [file pone.0008821.s005.tif]

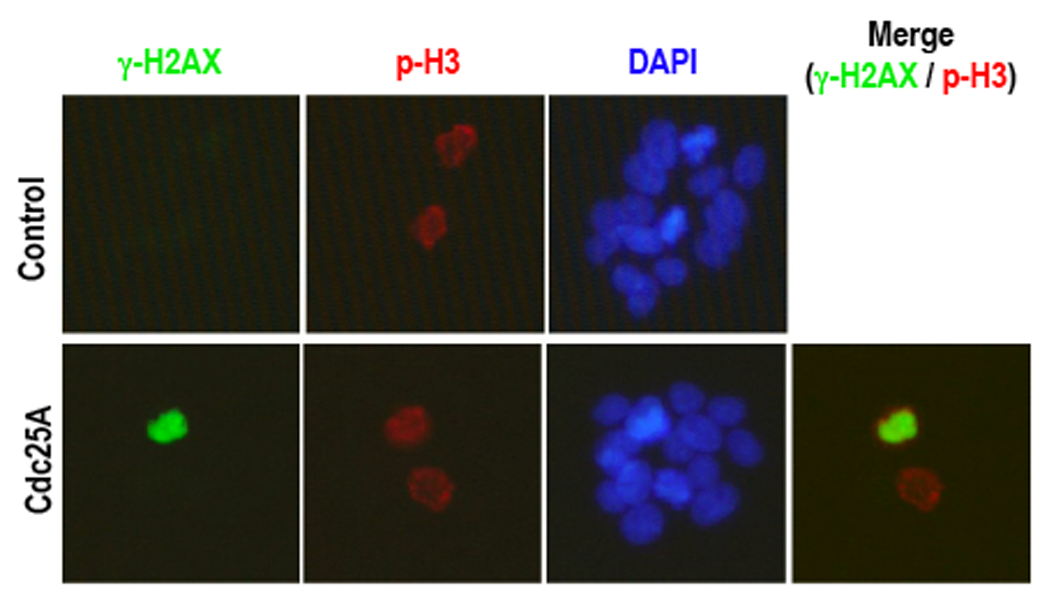

Supplement: Figure S2 — Transient over-expression of Cdc25A promotes DNA lesions including the cells during mitosis. Empty (control) or Cdc25A expression (Cdc25A) vectors were transfected into HEK293 cells. After cultivation for two days, cells were determined with the indicated antibodies. (3.02 MB TIF) [file pone.0008821.s006.tif]

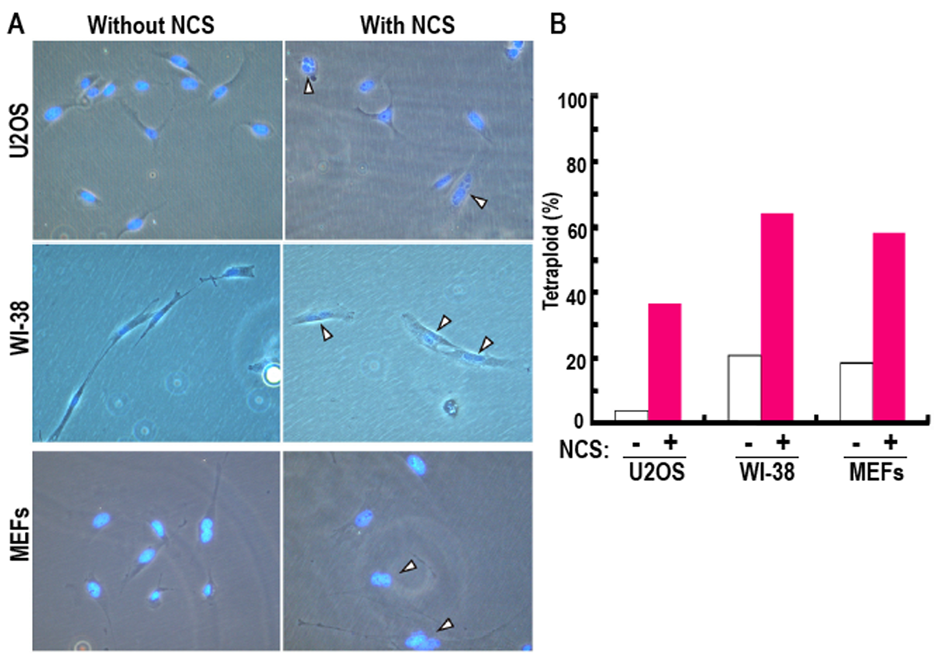

Supplement: Figure S3 — Tetraploidy generation with DNA damage during mitosis in U2OS, WI-38 and primary MEFs. A. Cells prepared as in the experimental scheme on Fig. 5A were stained with DAPI. The arrowheads indicate bi-nuclear tetraploid cells. B. Quantification of the tetraploid cells was performed with at least 100 cells for each. (2.99 MB TIF) [file pone.0008821.s007.tif]

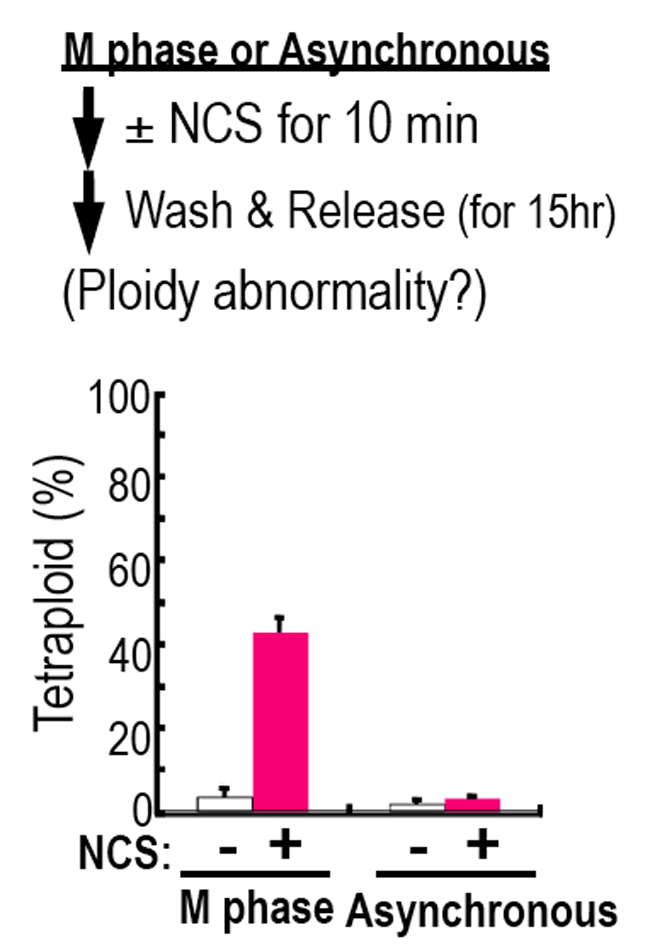

Supplement: Figure S4 — Cells damaged during mitosis lead to tetraploidy generation but not during interphase. HeLa cells in the M phase or without synchronization were treated as in the scheme. Unlike asynchronous cells, M phase-cells specifically develop tetraploidy after damage. Quantification of the tetraploid cells was performed with at least 100 cells for each. (2.21 MB TIF) [file pone.0008821.s008.tif]

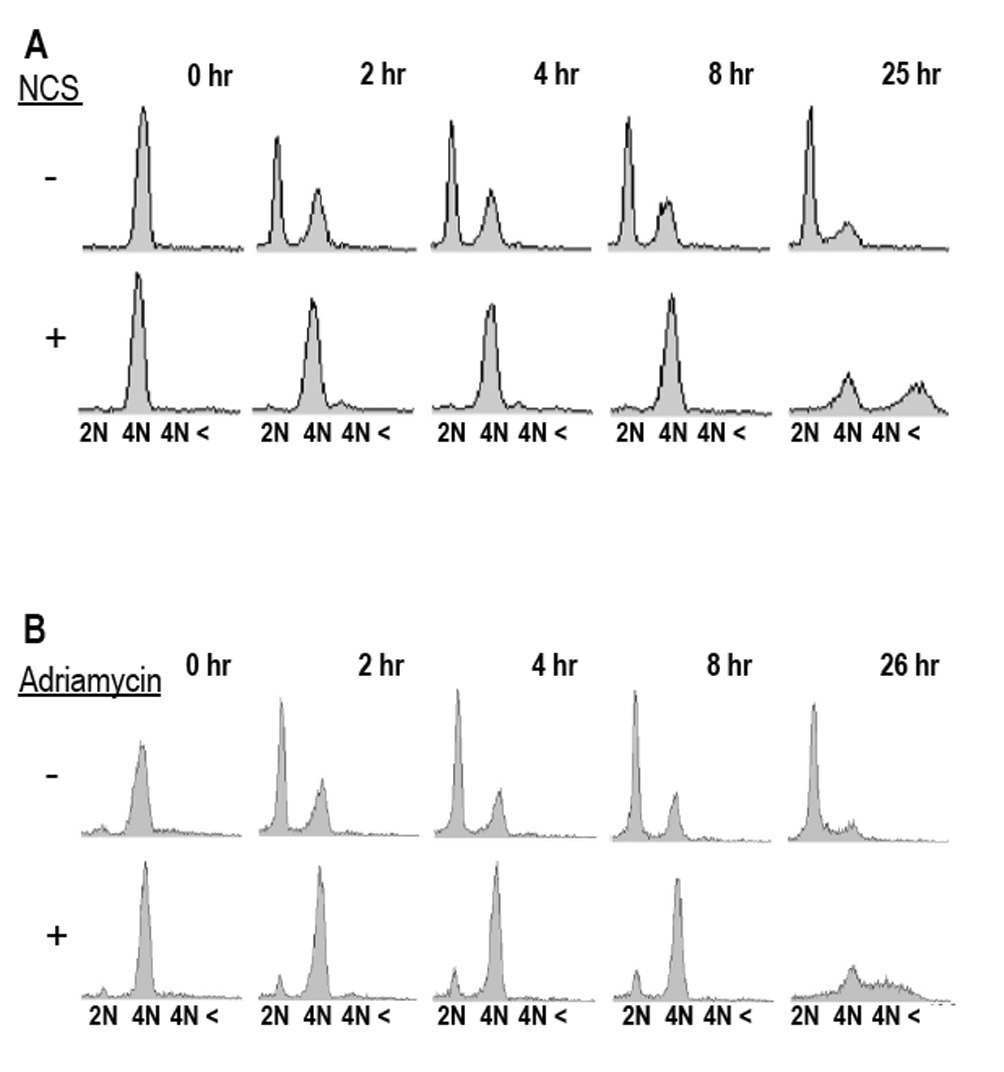

Supplement: Figure S5 — The cells damaged in the M phase further replicate DNAs in the following S phase. A,B. After cells were damaged with NCS (A) or adriamycin (B) as in Fig. 5A, the chromosome contents of the cells after the release were analyzed by flow cytometry. (1.10 MB TIF) [file pone.0008821.s009.tif]

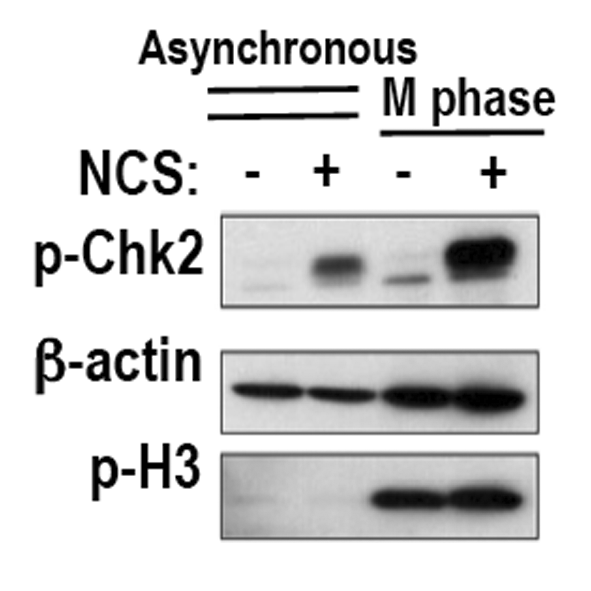

Supplement: Figure S6 — DNA damage checkpoint activation is durable in the M phase, but dysfunctional to induce arrest during mitosis. The activation of DNA damage checkpoint protein Chk2 in the HeLa asynchronous and M-phase cells characterized by phosphorylated histone H3 (P-H3) was analyzed for the phosphorylated form. (0.37 MB TIF) [file pone.0008821.s010.tif]

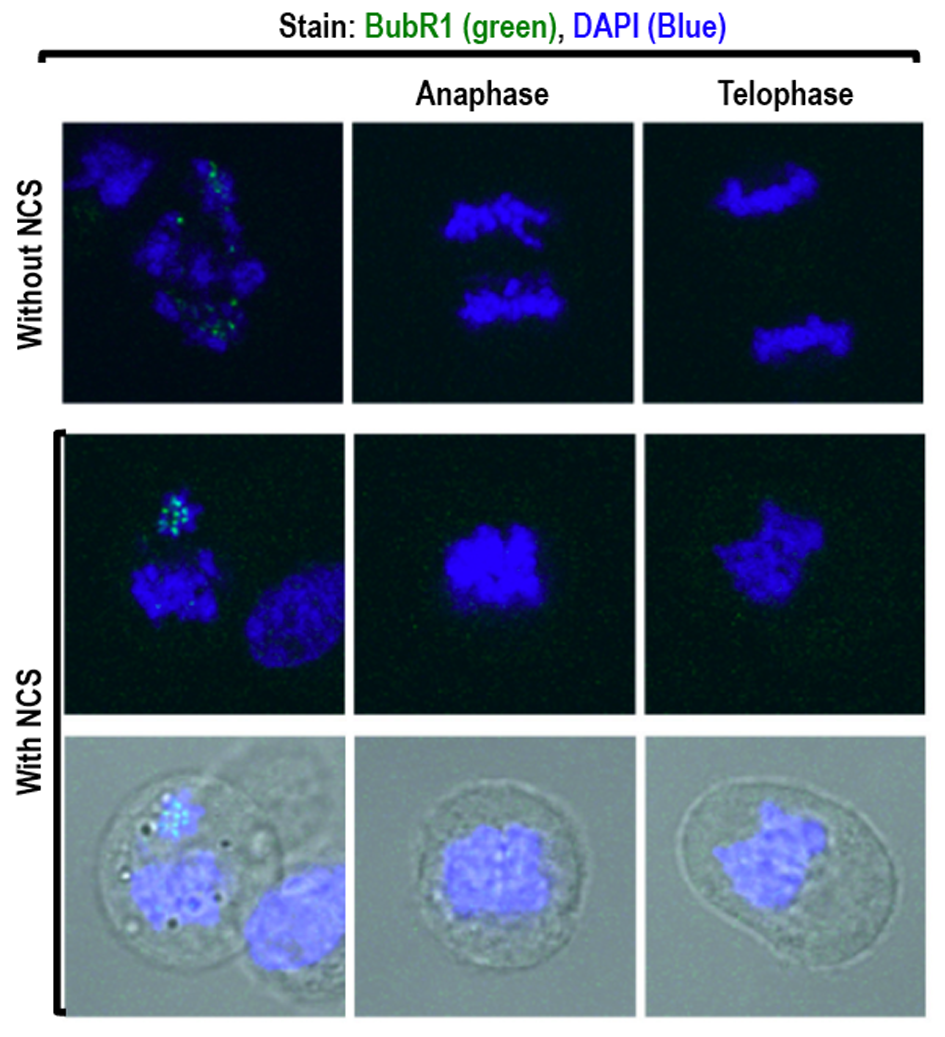

Supplement: Figure S7 — Prometaphase-DNA damage does not affect the behavior of BubR1 and the progression into the anaphase and the telophase. At 75 min after the release from NCS treatment as in the experimental scheme on Fig. 5A, the cells were stained with anti-BubR1 antibody and DAPI. For the NCS-treated cells, the mitotic stages in the anaphase and the telophase are estimated based on the degree of cell elongation. (4.78 MB TIF) [file pone.0008821.s011.tif]

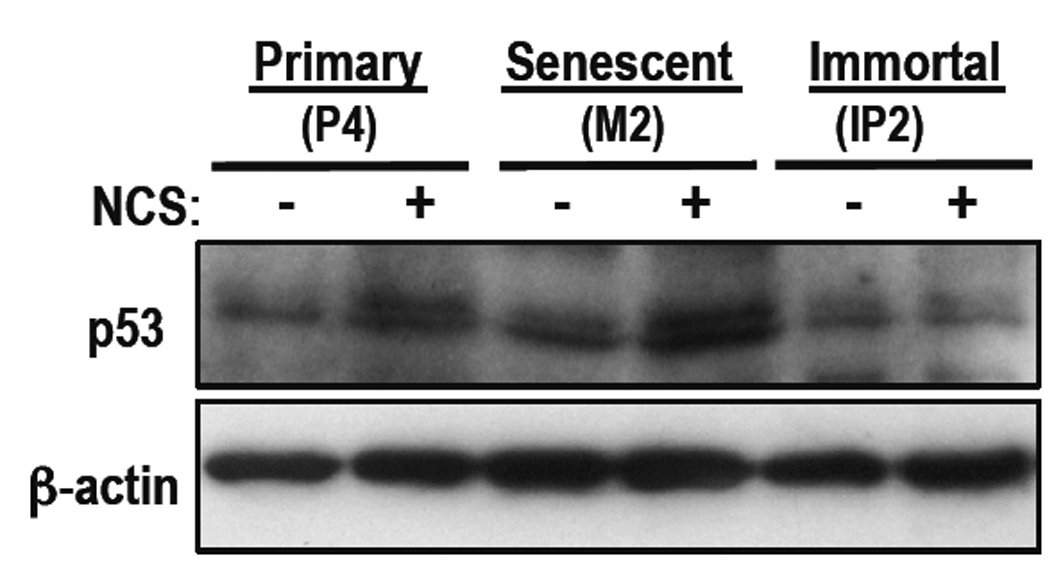

Supplement: Figure S8 — Arf/p53 module mutation in the immortalized MEFs. To determine the loss of Arf/p53 module, p53 accumulation was monitored 12 h after 100 ng/ml NCS treatment at each stage of MEFs: primary growth (P4); senescence (M2); immortalized (IP2). (0.63 MB TIF) [file pone.0008821.s012.tif]

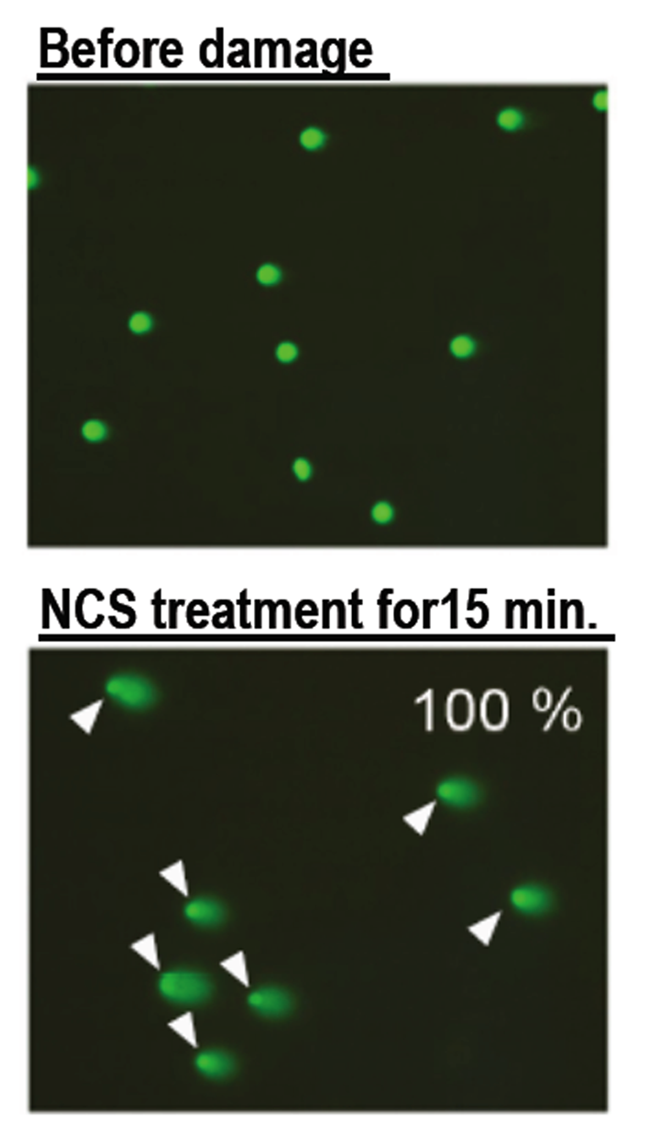

Supplement: Figure S9 — DNA lesions indicated by γH2AX were also confirmed with comet assay. DNA lesions, indicated by γH2AX in this study, were also confirmed by comet assay with the tails after NCS treatment for 15 min. Arrow heads indicate the spots with comet tails, indicating DNA damages. (2.88 MB TIF) [file pone.0008821.s013.tif]
